# Detection of *Coxiella burnetii* in Bulk Tank Milk of Dairy Small Ruminant Farms in Greece

**DOI:** 10.3390/foods14030460

**Published:** 2025-01-31

**Authors:** Daphne T. Lianou, Themistoklis Giannoulis, Charalambia K. Michael, Natalia G. C. Vasileiou, Efthymia Petinaki, Angeliki I. Katsafadou, Antonis P. Politis, Dimitris A. Gougoulis, Vasileios G. Papatsiros, Elias Papadopoulos, Nikolaos Solomakos, Eleni I. Katsarou, Vasia S. Mavrogianni, Dimitriοs C. Chatzopoulos, George C. Fthenakis

**Affiliations:** 1 Veterinary Faculty, University of Thessaly, 43100 Karditsa, Greece; dlianou@vet.uth.gr (D.T.L.); nsolom@uth.gr (N.S.);; 2 Faculty of Animal Science, University of Thessaly, 41110 Larissa, Greece; thgianno@uth.gr (T.G.);; 3 School of Veterinary Medicine, European University of Cyprus, Engomi, 2404 Nicosia, Cyprus; 4 University Hospital of Larissa, 41110 Larissa, Greece; 5 Faculty of Public and One Health, University of Thessaly, 43100 Karditsa, Greece; 6 Laboratory of Parasitology and Parasitic Diseases, School of Veterinary Medicine, Faculty of Health Sciences, Aristotle University of Thessaloniki, 54124 Thessaloniki, Greece; eliaspap@vet.auth.gr

**Keywords:** bulk tank milk, *Coxiella burnetii*, goat, qPCR, predictors, prevalence, Q fever, sheep, small ruminants

## Abstract

The objectives of this work were as follows: (i) the evaluation of the prevalence of detection of genetic material of *Coxiella burnetii* in the bulk tank milk of sheep and goat farms in Greece and (ii) the investigation of variables related to the management applied in farms as possible predictors for this. The presence of *C. burnetii* genetic material was studied in the bulk tank milk of 325 sheep and 119 goat farms throughout the country. For qualitative and quantitative identification of the genetic material of the pathogen, a commercially available real-time PCR was used. In total, 45 parameters were assessed for potential association with the detection of the pathogen: these referred to the management system, infrastructure, health management, animals, production characteristics, and human resources on the farms. Genetic material of the pathogen was detected in bulk tank milk samples from nine sheep (2.8%) and six goat (5.0%) farms. Genetic material was at significantly higher median concentrations in samples from goat farms than from sheep farms, 1,078,096 (min: 181,121, max: 2,331,386) versus 15,728 (min: 507, max: 505,852) GE mL^−1^, respectively. For sheep farms, the intensive or semi-intensive management system applied in farms (*p* = 0.003), and for goat farms, the intensive or semi-intensive management system applied in farms (*p* = 0.0007) and the smaller number of annual veterinary visits to farms (*p* = 0.044) emerged as significant predictors. Among sheep farms managed under the intensive or semi-intensive system, the lack of accessory barns on farms (*p* = 0.024) emerged as a significant predictor; no significant predictor could be found among goat farms under such management systems. There was no significant difference in production outcomes between farms in which *C. burnetii* was or was not detected in the bulk tank milk; also, there was no association between the detection of *C. burnetii* and the annual incidence rate of cases of abortion on the farms. The results suggest that the risk of transfer of *C. burnetii* to dairy products from sheep and goat milk appears to be small, but not negligible, which indicates that the pasteurization of milk from small ruminants must be carried out consistently and correctly to ensure the safety of the product.

## 1. Introduction

*Coxiella burnetii*, the causal agent of Q fever, can infect a variety of animal species and humans [1,2,3]. Domestic ruminants are the primary animal reservoir of the pathogen [3]. Infected animals may shed the organism mostly in genital excretions, as well as in feces and milk [4,5,6].

People are particularly susceptible to *C. burnetii*, given that as few as 10 cells of the organism can cause infection [7]. The vast majority of human cases occur subsequently to transmission of the pathogen from animals; indeed, transmission of the pathogen between people is rare [8]. Two major routes of human infection are prominent: airborne dissemination of the pathogen within or from animal farms and consumption of contaminated food (mainly milk) [9]. Transmission through a tick infestation is also a considerable possibility [10]. In humans, Q fever is a zoonotic infection, problematic in its diagnosis. This is due to many reasons; these include the variety of uncharacteristic clinical symptoms, which make its distinction from other diseases difficult, and the often negative results of diagnostic tests, which are based on antibody detection. In most cases, the infection is self-limiting [11,12,13]. The excretion of the pathogen in the milk of animals on a farm can lead to its accumulation in the bulk tank milk of the farm, where it can subsequently be transferred to the dairy products manufactured from this milk. A potential route of transmission of *C. burnetii* to humans is the consumption of contaminated raw milk or dairy products manufactured from such milk. There is epidemiological evidence that a proportion of cases of Q fever in people have arisen as a consequence of the consumption of milk or cheese containing the organism [13].

Field studies on the molecular detection of *C. burnetii* in bulk tank milk from small ruminant farms have been published from eight countries (Bulgaria, Iran, Italy, Poland, Portugal, Spain, Switzerland, the Netherlands, Türkiye). Among these, nine studies were performed on sheep farms and seven studies were performed on goat farms [14,15,16,17,18,19,20,21]. The median numbers of farms included in these studies were 81 sheep farms and 43 goat farms. The median prevalence among these studies was found to be 7.7% for sheep farms and 5.0% for goat farms (Table S1).

Greece is unique among European countries in its production of larger amounts of milk from small ruminants than from cows, annually [22,23]. In Greece, small ruminant farming is the most important agricultural animal farming business; it generates almost 18% of the total primary sector income and approximately 1% of the total annual gross domestic product in the country [24]. Milk production from sheep and goats is considered to be the highest among European countries [23]. In 2023, the total production by dairy companies amounted to approximately 730,000 metric tons of sheep milk and 160,000 metric tons of goat milk [25]. Milk produced from sheep or goats in Greece is used mainly for the production of cheese and yogurt, whilst a smaller quantity of caprine milk is consumed as fresh milk [23]. In 2022, the total cheese production from sheep and goat milk amounted to approximately 215,000 metric tons; most of that quantity (68–70%) was designated as ‘feta’ cheese, which is a recognized Protected Designation of Origin product within the European Union [26]. Over 65% of the feta cheese produced in the country is exported, with the total relevant revenue nowadays exceeding one billion euros annually [27]. Although the manufacture of cheese from unpasteurized milk is not allowed under the current legal provisions, the homemade small-scale production of traditional cheese based on this practice cannot be ruled out (e.g., Arseniko cheese produced on the island of Naxos [28]).

There is a clear zoonotic risk of the presence of *C. burnetii* in milk. A comprehensive literature review (2018) concluded that the risk of human infection by *C. burnetii* through the consumption of unpasteurized milk and of dairy products manufactured from such milk ‘cannot be considered negligible’ (*sic*) [10]. In Greece, a study into Q fever showed that children aged 11 to 14 years and children who had consumed cheese produced in rural areas of the country were at higher risk of developing the infection [29]. Overall, in the European Union, the total number of confirmed human cases of the infection is 0.19 cases per 100,000 people [30]. Nevertheless, the possible presence of *C. burnetii* in the milk produced in small ruminant farms in Greece has not been investigated before.

Therefore, there is an interest in studying the presence of *C. burnetii* in the bulk tank milk of sheep and goat farms. This would provide information on the presence of an important zoonotic pathogen therein. The study was carried out as part of a large country-wide mapping of the sheep and goat industry in the country. The specific objectives of the present work were (i) the evaluation of the prevalence of genetic material of *Coxiella burnetii* in the bulk tank milk of sheep and goat farms in Greece and (ii) the investigation of variables related to the management applied in farms as possible predictors for this presence.

## 2. Materials and Methods

### 2.1. Visits to Farms and Collection of Samples

**Figure 1:**
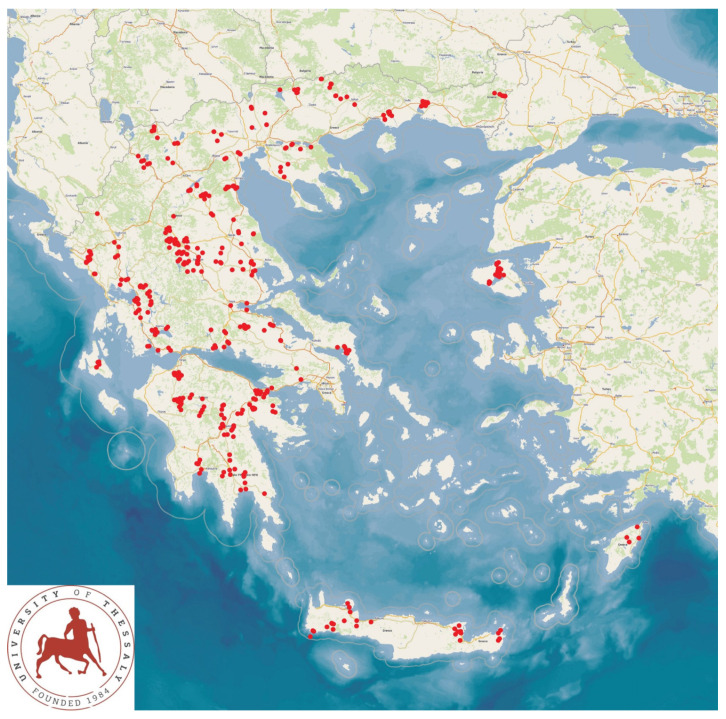
Locations of the 444 small ruminant farms across Greece (red dots), wherein the presence of *C. burnetii* genetic material was studied.

An extensive cross-sectional study was performed in dairy small ruminant farms in Greece. Visits were made to sheep (*n* = 325) and goat (*n* = 119) farms, which were located throughout the country, specifically in all the 13 administrative regions (Figure 1). The farms had been selected by collaborating veterinarians on a convenience basis (i.e., the willingness of farmers to accept a visit from university personnel for the collection of samples and to obtain relevant information). The investigators (D.T.L., C.K.M., and G.C.F.) visited all the farms in the study for sample collection.

The mean number of female animals in the farms into the study was 325 ± 13 ewes for sheep farms and 237 ± 20 does for goat farms [31]. The mean annual milk quantity produced per animal was 207 ± 5 L for sheep farms and 201 ± 10 L for goat farms [31]. In all farms where this was legally required, vaccination against *Brucella melitensis* infection was applied [31]. Overall, the reported incidence rate of abortion during the season preceding the visit was 2.0% (95% confidence interval: 1.9–2.1%) and 2.5% (95% confidence interval: 2.7–2.9%) for sheep and goat farms, respectively [31]. Median month into lactation at the sampling of animals on the visited farms was 5 (interquartile range: 4) [31].

During the visit, four 20 mL samples were collected from the bulk tank milk of each farm. The milk samples were collected directly from the cooling tank of each farm. The milk within each tank was stirred continuously and up until the opening of the tank cover for collection of the samples. These samples were obtained by means of sterile plastic single-use pipettes, which were immersed into the tank to withdraw milk. Two of the samples collected were to be used for cell counting and composition measurement (these were handled with no specific aseptic conditions), whilst the other two samples were to be used for the detection of pathogens (these were handled aseptically in all procedures).

Subsequently, an interview was carried out with the farmers, using a structured questionnaire (defined as “a document used to collect data from respondents, consisting of a set of standardized questions with a predetermined framework that set the precise language and sequence of the questions” without considering the respondents attitudes, personal opinions and values [32]). The document included pre-worded questions regarding the management system applied in the farm (*n* = 1), the farm infrastructure (*n* = 5), the health management practices carried out and applied in the farm (*n* = 23), the animal populations on the farm (small ruminants or other domestic animals) (*n* = 7), the production outcomes (*n* = 3), and the human resources, including the socio-demographic characteristics of the farmers (*n* = 6) [31]. The details are shown in Table S2.

The milk samples were stored at 0.0 to 4.0 °C using ice packs in portable refrigerators.

### 2.2. Sample Processing and DNA Extraction

Initially, milk composition measurement and somatic cell counting were performed on each of the two relevant samples within 4 h of sample collection, as detailed before [31]. Transportation of samples to the laboratory was then carried out by the investigators by car; samples collected from farms on the islands were also transported as ice-packed packages, accompanying luggage by airplane or by boat.

The two samples for the detection of pathogens were initially used for bacteriological examinations [31]; then, they were mixed to form one sample from each farm. This sample was centrifuged at 1000× *g* for 10 min and thereafter the supernatant was discarded, whilst the pellet obtained was resuspended in phosphate-buffered saline, pH 7.3, and used for DNA extraction. This was performed by means of a commercially available kit (IndiSpin Pathogen Kit; Indical BioScience, Leipzig, Germany), following the manufacturer’s protocol.

The quality and the quantity of the extracted DNA was assessed using agarose gel electrophoresis and photometry by means of a Nanodrop, respectively. To confirm the extraction of bacterial DNA, a quantitative PCR (qPCR) was performed, using a primer pair that targets the hypervariable region V3–V4 of the 16s rRNA gene (EUB 338f-518r). qPCR was performed using SYBR Green technology with a RotorGene Q (Qiagen; Hilden, Germany). Analysis of the qPCR was performed in a 20 μL reaction volume by adding 0.1 ng of DNA in the PCR mix containing gene specific primers (200 nM final concentration) and 1× KAPA SYBR FAST qPCR Master mix (Sigma-Aldrich; Saint Louis, MI, USA). The qPCR conditions were 5 min at 95 °C for denaturation and 40 cycles of denaturation (20 s at 95 °C) and annealing/extension (20 s at 60 °C).

### 2.3. Detection of C. burnetii Genetic Material

For the qualitative and the quantitative identification of *C. burnetii* genetic material, a commercially available test (bactotype *C. burnetii* PCR Kit; Indical BioScience, Leipzig, Germany) was used. The test was performed according to the manufacturer’s protocol. The DNA standard that contained *C. burnetii* DNA was serially diluted to make a standard curve for the quantification of the positive samples. The qPCR was performed in a RotorGene Q (Qiagen; Hilden, Germany), in a 25 μL reaction volume, by adding 5 μL of the extracted DNA and 20 μL of the master mix. qPCR conditions were set as follows: an initial denaturation step for 5 min at 95 °C, followed by 40 cycles of a denaturation step (10 s at 95 °C), and an annealing/extension step (30 s at 57 °C). Fluorescence collection was performed on the second step of the reaction. The quantity of *C. burnetii* DNA, expressed in genome copies, was calculated according to the manufacturers’ protocol by using the Cq values and the standard curve.

### 2.4. Data Management and Analysis

The detection of *C. burnetii* genetic material in a milk sample during the qPCR analysis was deemed to indicate the presence of the organism in the bulk tank milk of the farm.

All the data were systematically recorded and organized using Microsoft Excel (versions 1901-2412) (Microsoft Corporation, Redmond, WA, United States of America). Initially, basic descriptive analyses were performed.

The following two outcomes were considered: ‘detection of *C. burnetii* genetic material in the bulk tank milk from sheep/goat farms’ and ‘detection of *C. burnetii* genetic material in the bulk tank milk from sheep/goat farms under intensive or semi-intensive management’. In total, 45 and 44 parameters, respectively, from the information obtained during this study (Table S2) were evaluated for potential association with each of the above outcomes in a series of univariable analyses by using Spearman’s rank correlation analysis. The management system applied in farms was classified as intensive, semi-intensive, semi-extensive, or extensive in accord with the system described by the European Food Safety Authority [33]. Then, multivariable analysis was performed using mixed-effects logistic regression, with farms as the random effect. All variables with *p* < 0.2 in the univariable analyses were offered to the model. This was followed by a progressive removal of variables, and the ones included in the final multivariable models constructed are shown in Table S3. Separate analyses were performed for the sheep and goat farms.

Production and health outcomes were compared by using the Mann-Whitney test between farms where the pathogen was or was not detected in the bulk tank milk.

A *p* value below 0.05 (<5.0%) was considered to be statistically significant.

## 3. Results

### 3.1. Detection of Genetic Material of C. burnetii in Bulk Tank Milk

**Figure 2:**
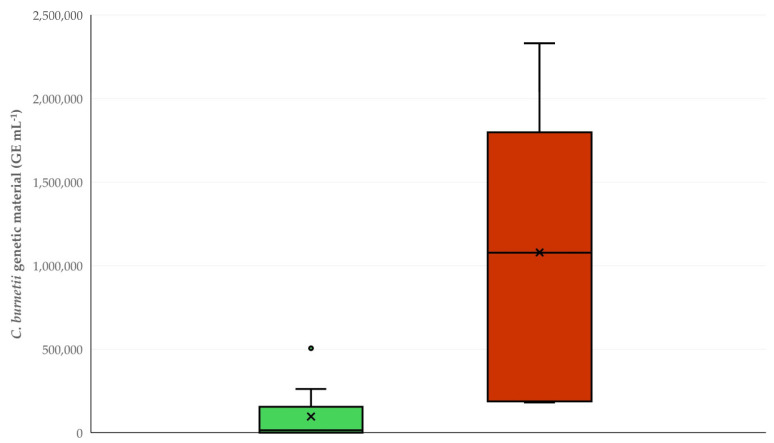
Box and whisker plot of the concentrations of *C. burnetii* genetic material detected in the bulk tank milk of sheep (green) or goat (brown) farms (GE: genome equivalents).

**Figure 3:**
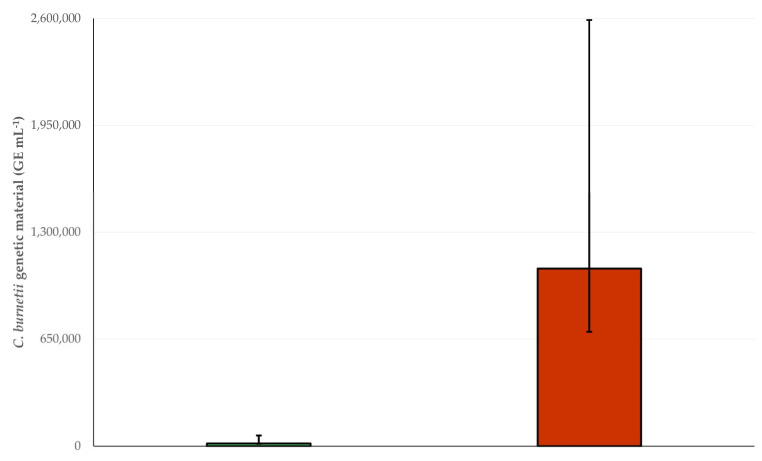
Median concentrations of *C. burnetii* genetic material detected in the bulk tank milk of sheep (green) or goat (brown) farms (bars indicate interquartile range; GE: genome equivalents).

In total, *C. burnetii* genetic material was detected in bulk tank milk using the Taqman assay in samples from fifteen (15) farms (3.4% (95% CI: 2.1–5.5%)). Specifically, *C. burnetii* genetic material was detected in samples from nine (9) sheep farms (2.8% (95% CI: 1.5–5.2%)) and six (6) goat farms (5.0% (95% CI: 2.3–10.6%)). There was no significant difference in the prevalence of detection of *C. burnetii* genetic material between sheep and goat farms (*p* = 0.24). However, genetic material was at significantly higher median concentrations in samples from goat farms than from sheep farms: 1,078,096 (interquartile range: 1,126,794; min: 181,121, max: 2,331,386) GE mL^−1^ versus 15,728 (interquartile range: 48,010; min: 507, max: 505,852) GE mL^−1^, respectively (*p* = 0.005) (Figure 2 and Figure 3) (GE: genome equivalents).

**Figure 4:**
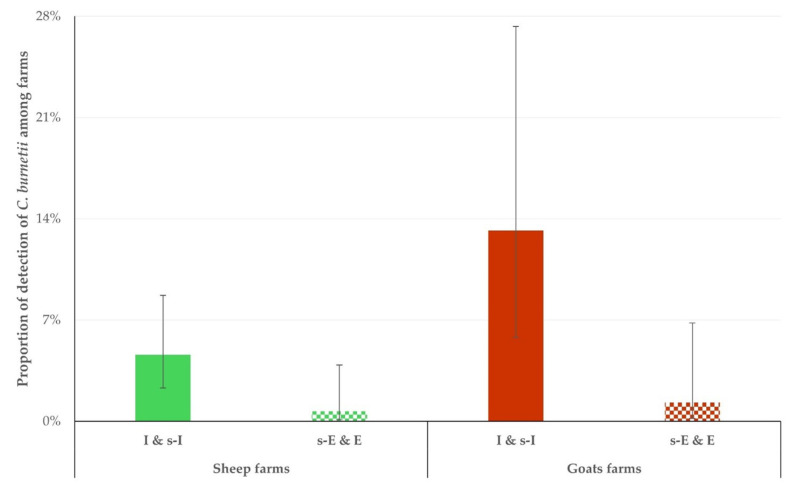
Proportion of farms in which *C. burnetii* genetic material was detected in bulk tank milk, in accord with the management system applied on the farms (intensive or semi-intensive: full bars, semi-extensive or extensive: motif bars).

*C. burnetii* genetic material was detected in significantly more samples from farms that applied the intensive or semi-intensive management system rather than in samples from farms that applied the semi-extensive or extensive management system: in thirteen (13) (5.9% (95% CI: 3.5–9.8%)) versus two (2) (0.9% (95% CI: 0.3–3.2%)) farms overall (*p* = 0.004). Similar findings were seen for sheep (eight (8) and one (1) farm, respectively) and goats (five (5) and one (1) farm, respectively) (*p* < 0.048 for both comparisons) (Figure 4).

The overall proportion of farms in which *C. burnetii* genetic material was detected was highest in the administrative regions of North Aegean (9.1%), Crete (6.9%), and Thessaly (5.6%).

### 3.2. Predictors

The results of the univariable analyses are in Tables S4–S7.

During multivariable analysis, the following variables emerged as significant predictors for the detection of *C. burnetii* in the bulk tank milk. For sheep farms, only the management system (intensive or semi-intensive) applied in farms (*p* = 0.003) emerged as a significant predictor. For goat farms, (i) the management system (intensive or semi-intensive) applied in farms (*p* = 0.0007) (Figure 4) and (ii) the smaller number of annual veterinary visits to farms (*p* = 0.044) emerged as significant predictors. Details are in Table 1.

Further, the following variables emerged as predictors for the detection of *C. burnetii* in the bulk tank milk of farms, where intensive or semi-intensive management was applied. For sheep farms, only the lack of accessory barns on the farms (*p* = 0.024) emerged as a significant predictor (Table 2). For goat farms, no significant predictors emerged; a tendency was found in the multivariable analysis for the concurrent presence of pigs on farms (*p* = 0.09) as a predictor.

### 3.3. Associations with Production- and Health-Related Outcomes

No significant differences were seen among the production-related outcomes of bulk tank milk between farms in which *C. burnetii* was or was not detected (for all comparisons, *p* > 0.47 for sheep farms and *p* > 0.32 for goat farms) (Table S8).

Moreover, no significant difference was seen in the annual incidence rate of abortion cases between farms in which *C. burnetii* was or was not detected (*p* = 0.76 for sheep farms and *p* = 0.93 for goat farms). Finally, no difference was found in the proportion of farmers who declared abortion as an important health problem in their farms, in accordance with the detection of *C. burnetii* (*p* > 0.41 for all comparisons) (Table S9).

## 4. Discussion

### 4.1. Presence of C. burnetii in Bulk Tank Milk

The current study explored the presence of *C. burnetii* in the bulk tank milk of sheep and goat farms in Greece. In relation to studies performed in other countries, this work included a higher number of farms and also country-wide geographical coverage. In this way, possible regionally important factors weighed less. In general, the results are similar to those of studies performed in other countries (i.e., with the prevalence of the presence of *C. burnetii* being <10%) and suggest that there is a small risk of transfer of the organism to the food chain. This may potentially reflect a low-level infection among small ruminants in Greece.

In general, the findings of the present study indicate that, in general, the prevalence of the pathogen in bulk tank milk is smaller than that detected in studies performed in other European countries. This refers to both sheep (Switzerland: 0.0% [14], Portugal: 1.3% [21], Bulgaria: 7.7% [20], Türkiye: 16.7% [17], Spain: 22.0% [15], Poland: 22.2% [18], Italy: 23.9% [19]) and goat (Bulgaria: 0.0% [20], Switzerland: 0.0% [14], Türkiye: 25.0% [17], The Netherlands: 33.0% [16], Poland: 51.2% [18]) farms.

A direct comparison of the findings recorded in the various countries is difficult. The differences may possibly reflect variations in the methodologies applied during the field work rather than true differences between countries. The present study included farms with all types of management systems, covered a wide geographical distribution, and contained farms with a wide range of lactation stages. In contrast, the smaller number of farms included in other studies (where the median numbers of farms included in those studies were 81 sheep and 43 goat farms) might have contributed to biased results; moreover, in other studies, sampling might have been carried out nearer the lambing/kidding period, when contamination of the farm environment from pathogens disseminated during parturition would have been higher. The results may also reflect differences in the management systems and practices applied in the various countries, which might also have accounted for the differences; for example, the higher proportion of farms under semi-extensive or extensive management systems in Greece could have contributed to the lower prevalence of the presence of *C. burnetii*. Further, one should also take into account climatological conditions that prevail at the various locations of the farms, as the airborne dissemination of the pathogen is facilitated by winds occurring there. Last, the differences may reflect a better management and greater care from the part of all involved in sheep and goat production in Greece (technical persons, as well as shepherds and goatherds), because of the importance of small ruminant dairy production in the country.

In a field study performed previously in the region of Thessaly (where one of the highest proportions of farms with presence of the pathogen in bulk tank milk was seen in the present study, 5.6%), the prevalence of antibodies against *C. burnetii* among small ruminants was found to be 14.5% [34]. One may suggest that, in sheep, the prevalence of antibodies against the pathogen in individual animals would be higher than its prevalence in the bulk tank milk. Sheep shed the pathogen mostly through faeces and vaginal mucus and less often in milk. In contrast, goats excrete the organism in milk, vaginal mucus, and faeces [35,36]. This is reflected in the current findings of smaller concentrations of the pathogen in the bulk tank milk of sheep farms; the present findings corroborate the experimental results of Bauer et al. [36] through the present large-scale field work.

Q fever in sheep is considered to be a benign disease [37], sporadically causing abortions [38] and developing mainly as a subclinical infection [39]. In goats, the infection has been described as a mild disease that develops with the final stage of gestation abortions and stillbirths [40,41]. The lack of differences in the incidence rates of abortion cases between farms according to presence or no presence of the pathogen in the bulk tank milk reflects the above.

### 4.2. Molecular Detection of C. burnetii

A real-time PCR method was employed in the present work, using probes to ensure the sensitivity of the process. The method can detect the genetic material of the pathogen even at low concentrations (as low as hundreds of genome copies per mL). Further, and unlike ELISA, which detects host antibodies as an indirect indicator of infection, PCR detects the pathogen itself, making it more reliable for indicating shedding of the pathogen occurring among animals within a farm at the time of sampling, and additionally also potential environmental contamination of the milk produced at the farm [42]. Moreover, DNA is a stable biomolecule, so the various storage methods (and their duration) used for the milk to be examined would not affect the detection of genetic material to the same extent as in other molecules (e.g., RNA or proteins), which have also been used in various protocols for the identification of *C. burnetii* [43,44]. With regard to diagnosis of the infection in individual animals, the technique is particularly useful for use during the acute phase of infection i.e., before the immune system starts producing detectable antibody levels, on which ELISA is based and relies on; hence, using ELISA on milk for the detection of the pathogen may miss early stages infections or cases of low antibody titers [42,45].

PCR is effective in the detection of *C. burnetii* genetic material, but it also has some relevant limitations. Among them is the difficulty to differentiate, due to its nature, between current (active) and past infections when used in diagnostic procedures [46]. Further, false-negative results may possibly arise from the presence of PCR inhibitors in clinical specimens, for example, blood or tissues, which may inhibit amplification [47]. Moreover, the high sensitivity of the technique also creates a risk in case of contamination, which would result in false-positive results. Therefore, and given that PCR has a restriction in the level of genetic information garnered, subsequent locus detection systems (e.g., multi-locus sequence typing) are useful for differentiation between strains [48].

In order to minimize the adverse effects of the above issues, a commercial kit was used for the molecular detection of the pathogen. The kit has a specific design that utilizes two sets of primers and probes; one of these acted as an internal control, reducing false-positive results. Further, the use of negative controls in each reaction during testing ensured the avoidance of false-negative results.

### 4.3. Predictors

The identification of intensive or semi-intensive management systems as the primary predictor for the presence of *C. burnetii* in the bulk tank milk of a farm reflects the mode of transmission of the pathogen in small ruminant farms. This type of management involves the housing of animals for a significant period of time daily [33], which facilitates transmission of the pathogen between animals. Inhalation of aerosolized *C. burnetii*, which can occur more easily among housed animals, is a common means of animal infection [3,49]. Subsequently, animal infection is followed by the pathogen’s potential excretion in milk. Direct contamination of the bulk tank milk with *C. burnetii* aerosols, present within the farm buildings, may also occur and should not be ruled out. In contrast, sheep and goats managed under a semi-extensive or an extensive system are housed less often and for smaller time periods; this contributed to the significantly less frequent presence of the pathogen in this bulk tank milk.

The homemade production of cheese takes place more often in farms managed under the semi-extensive or extensive systems (in 75.7% of farms versus in 56.3% of farms managed under the intensive or semi-intensive system [31]). It may be postulated that the rare presence of the pathogen in the bulk tank milk of farms managed semi-extensively or extensively significantly reduces the potential for transfer of the pathogen into the food chain through the processing of unpasteurized milk. Farms under the intensive or semi-intensive systems, where the presence of the pathogen was more frequent, deliver their milk to dairy companies, which follow appropriate measures for hygiene processing of the raw milk.

The transmission of the pathogen among small ruminants is facilitated in animal houses with dusty and dry environments [50], conditions which prevail in barns with high animal concentrations. The presence of accessory barns and houses on a farm would lead to smaller animal concentrations therein, thus reducing the potential risk of infection of the animals. Hence, the lack of accessory barns as a predictor for the presence of the pathogen is fully in line with the above. The presence of only one animal house on a farm can lead to higher animal concentration within that building. In turn, this contributes to increased pathogen circulation and thus to the presence of the organism in milk.

Recognizing the presence of pigs on farms as a predictor for the detection of *C. burnetii* in milk was an unexpected finding. In general, there is little knowledge regarding *C. burnetii* infection of pigs; it is unclear whether pigs can transmit the organism and whether *Coxiella* infection of pigs can significantly impact their productivity or public health [51,52,53], although it was described that shedding of the organism can occur from all domesticated species [50]. The results of a recent field study performed in Southern Italy indicated that the prevalence of *C. burnetii* infection among pig farms located in areas with a high density of ruminants was 4.2%, higher than among pig farms away from ruminant farms [54]. That region shares a similar, Mediterranean-type climate with Greece, which supports the epidemiological cycle of pathogens through vectors, including ticks (e.g., *Ixodes* spp., *Rhipicephalus* spp., which can be found on both pigs and small ruminants [55,56,57]), thus potentially playing a role in the transmission of *C. burnetii* [53] and its consequent excretion in milk. Moreover, pigs scavenge within farms and thus might disseminate the pathogen, which can contribute to the infection of small ruminants [50]. The presence of pigs on farms, animals which scavenge within the area of a farm, can contribute to the development and emergence of aerosols. The regional landscape and climatic conditions prevailing in the dry, Mediterranean-type climate present at the locations of the sheep and goat farms in Greece can further contribute to the dissemination of these aerosols within and away from the farm, and consequently to the contamination of the bulk tank milk of the farms [58,59].

The co-existence of pigs with small ruminants was recorded in 11.3% of sheep and goat farms in Greece [31]. Further, for pathogens for which transmission from one species to another (e.g., foot-and-mouth disease virus) is well-documented, it is noted that in a previous study, we identified the presence of pigs as a predictor for the isolation of *Listeria* spp. from the bulk tank milk of sheep farms [31]. These findings indicate that in farms where pigs are present, increased biosecurity measures must be implemented to avoid the transfer of pathogens between animal species. The possibility of pathogen transfer from wild boar roaming in the vicinity of the farms to domestic pigs on the farms and then to small ruminants should also be taken into account.

### 4.4. The Importance of Milk Pasteurization for the Prevention of Milk-Borne C. burnetii Human Infections

Since the pasteurization of milk prior to human consumption or processing has become mandatory, a paramount decrease in the incidence of milk-borne diseases has been recorded around the world [60]. In the United States of America, during the period of 1993 to 2006, outbreaks of human infections subsequent to the consumption of unpasteurized dairy products were estimated to be almost 150 times higher (per unit of product) than outbreaks associated with the consumption of pasteurized dairy products [61]. Raising awareness about the importance of milk pasteurization can lead to informed consumer choices regarding the health risks from raw milk consumption and can promote safe milk-handling practices.

*C. burnetii* is resistant and can survive at 70 °C for 15 min. The pathogen is considered to be the most heat-resistant non-spore-forming organism in milk; thus, populations of the pathogen must be reduced by at least five log_10_-steps during the pasteurization procedure [62]. A study by Enright et al. [63] was the first to suggest that a high temperature (>70 °C) was needed to destroy this organism in milk [64], although the existence of *Coxiella* isolates resistant to such temperatures cannot be excluded [62]. Nevertheless, the decision to increase the pasteurization temperatures of milk in order to destroy *Coxiella* might be considered an early example of the application of the precautionary principle [65] and has contributed to minimizing milk-borne human cases of Q fever.

## 5. Conclusions

*C. burnetii* infection in small ruminants is considered to be rather mild [37,38]. However, the potential for transmission of the causal organism to people raises concerns. Consumption of unpasteurized milk that includes the pathogen is a confirmed means of human infection [41].

The present study provides the first information, based on country-wide data collection, about the presence of *C. burnetii* in the bulk tank milk of sheep and goat farms in Greece. The genetic material of the pathogen was detected in the bulk tank milk of 3.4% of 444 farms. It was detected more frequently and in higher concentrations in milk from goat farms than from sheep farms. The intensive and semi-intensive management systems emerged as the primary predictors for the detection of the pathogen in bulk tank milk, which may reflect the mode of transmission of the pathogen among small ruminants. The present large-scale results suggest that the risk of transfer of *C. burnetii* to dairy products from sheep and goat milk appears to be small, but not negligible, which indicates that the pasteurization of milk from small ruminants must be carried out consistently and correctly to ensure the safety of the product.

Measures to maintain *C. burnetii* infections at a low level should be continued in sheep and goat farms. At the farm level, these include the application of biosecurity measures to prevent introduction of the pathogen in farms, the implementation of appropriate health management to contain the pathogen, the investigation of cases of abortion for the early diagnosis of possible cases of the infection, and routine, periodic testing of the bulk tank milk of farms for detection of the pathogen. Such actions will contribute to further reducing the level of infection by the pathogen.

At the dairy factory level, it is imperative to correctly implement pasteurization procedures on raw milk, which will destroy the pathogen. All of the above measures will contribute to a high quality of dairy products from sheep and goat milk across the country.

With regard to human cases of the infection, the source of the pathogen in diagnosed cases of Q fever in people should be investigated to evaluate possible associations with dairy products. Subsequent tracing to the farm level will account for possible errors at the farm or dairy factory levels.

Future directions of this work could include the molecular typing of the isolates to study the dissemination patterns of the pathogen within the country, which would contribute to further controlling the infections at the farm level. Finally, the development of tools based on machine learning, in order to predict the presence of the pathogen in milk produced at the farms, can also be assessed.

## Figures and Tables

**Table 1:** Results of multivariable analysis for predictors for the detection of genetic material of *C. burnetii* in the bulk tank milk of small ruminant farms in Greece.

Variables	Odds Ratio (95% CI)/ Odds Risk (± se) ^1^	*p*
Sheep Farms		
Management system applied in farms		0.003
Intensive or semi-intensive (4.4% ^2^)	6.364 (0.787–1.486)	0.08
Semi-extensive or extensive (0.7%)	reference	-
Goat Farms		
Management system applied in farms		0.0007
Intensive or semi-intensive (4.4%)	12.121 (1.363–107.769)	0.025
Semi-extensive or extensive (0.7%)	reference	-
Annual number of veterinary visits to farms		0.044
Per unit (visit) decrease (*r_sp_* = −0.209)	1.006 ± 1.003	0.06

^1^ CI: confidence interval, se: standard error; ^2^ proportion of farms with the outcome under evaluation.

**Table 2:** Results of multivariable analysis for predictors for the detection of genetic material of *C. burnetii* in the bulk tank milk of sheep farms under intensive or semi-intensive management in Greece.

Variable	Odds Ratio (95% CI) ^1^	*p*
Availability of accessory barns on farms		0.024
Yes (77.8% ^2^)	reference	-
No (50.0%)	3.513 (0.840–14.692)	0.09

^1^ CI: confidence interval; ^2^ proportion of farms with the outcome under evaluation.

## Data Availability

The original contributions presented in this study are included in the article/Supplementary Materials. Further inquiries can be directed to the corresponding author.

## Supplementary Materials

The following supporting information can be downloaded at https://www.mdpi.com/article/10.3390/foods14030460/s1, Table S1: Prevalence of detection of *Coxiella burnetii* genetic material in the bulk milk tank of sheep and goat farms in accordance with published studies from countries around the world; Table S2: List of variables evaluated for potential association with the detection of *Coxiella burnetii* genetic material in the bulk tank milk of 325 sheep flocks and 119 goat herds in Greece; Table S3: Details of multivariable models employed for the evaluation of potential associations with the detection of *Coxiella burnetii* genetic material in the bulk tank milk of 325 sheep flocks and 119 goat herds in Greece; Table S4: Results of univariable analysis for the detection of *Coxiella burnetii* genetic material in the bulk tank milk of 325 sheep flocks in Greece; Table S5: Results of univariable analysis for the detection of *Coxiella burnetii* genetic material in the bulk tank milk of 119 goat herds in Greece; Table S6: Results of univariable analysis for the detection of *Coxiella burnetii* genetic material in the bulk tank milk of farms un-der intensive or semi-intensive management among 325 sheep flocks in Greece; Table S7: Results of univariable analysis for the detection of genetic material of *Coxiella burnetii* in the bulk tank milk of farms under intensive or semi-intensive management among 119 goat herds in Greece; Table S8: Production-related outcomes in farms where *Coxiella burnetii* was or was not detected in the bulk tank milk among 325 sheep flocks and 119 goat herds in Greece; Table S9: Health-related outcomes in farms where *Coxiella burnetii* was or was not detected in the bulk tank milk among 325 sheep flocks and 119 goat herds in Greece.

## References

[1] Babudieri B., Moscovici C. (1952). Experimental and natural infections of birds by *Coxiella burnetii*. Nature.

[2] Arricau-Bouvery N., Rodolakis A. (2005). Is Q fever an emerging or re-emerging zoonosis?. Vet. Res..

[3] Van den Brom R., van Engelen E., Roest H.I., van der Hoek W., Vellema P. (2015). *Coxiella burnetii* infections in sheep or goats: An opinionated review. Vet. Microbiol..

[4] Maurin M., Raoult D. (1999). Q fever. Clin. Microbiol. Rev..

[5] Arricau-Bouvery N., Souriau A., Lechopier P., Rodolakis A. (2003). Experimental *Coxiella burnetii* infection in pregnant goats: Excretion routes. Vet. Res..

[6] Wouda W., Dercksen D.P. (2007). Abortion and stillbirth among dairy goats as a consequence of *Coxiella burnetii*. Tijdschr. Diergeneeskd..

[7] Pexara A., Solomakos N., Govaris A. (2018). Q fever and prevalence of *Coxiella burnetii* in milk. Trends Food Sci. Tech..

[8] Ullah Q., Jamil T., Saqib M., Iqbal M., Neubauer H. (2022). Q Fever—A Neglected Zoonosis. Microorganisms.

[9] Bontje D.M., Backer J.A., Hogerwerf L., Roest H.I.J., van Roermund H.J.W. (2016). Analysis of Q fever in Dutch dairy goat herds and assessment of control measures by means of a transmission model. Pract. Vet. Med..

[10] Gale P., Kelly L., Mearns R., Duggan J., Snary E.L. (2015). Q fever through consumption of unpasteurised milk and milk products—A risk profile and exposure assessment. J. Appl. Microbiol..

[11] Klaassen C.H., Nabuurs-Franssen M.H., Tilburg J.J., Hamans M.A., Horrevorts A.M. (2009). Multigenotype Q fever outbreak, the Netherlands. Emerg. Infect. Dis..

[12] Polo M.F., Mastrandrea S., Santoru L., Arcadu A., Masala G., Marras V., Bagella G., Sechi M.M., Tanda F., Pirina P. (2015). Pulmonary inflammatory pseudotumor due to *Coxiella burnetii*. Case report and literature review. Microbes Infect..

[13] Sireci G., Badami G.D., Di Liberto D., Blanda V., Grippi F., Di Paola L., Guercio A., de la Fuente J., Torina A. (2021). Recent advances on the innate immune response to *Coxiella burnetii*. Front. Cell. Infect. Microbiol..

[14] Fretz R., Schaeren W., Tanner M., Baumgartner A. (2007). Screening of various foodstuffs for occurrence of *Coxiella burnetii* in Switzerland. Int. J. Food Microbiol..

[15] García-Pérez A.L., Astobiza I., Barandika J.F., Atxaerandio R., Hurtado A., Juste R.A. (2009). Investigation of *Coxiella burnetii* occurrence in dairy sheep flocks by bulk-tank milk analysis and antibody level determination. J. Dairy Sci..

[16] Van den Brom R., van Engelen E., Luttikholt S., Moll L., van Maanen K., Vellema P. (2012). *Coxiella burnetii* in bulk tank milk samples from dairy goat and dairy sheep farms in The Netherlands in 2008. Vet. Rec..

[17] Can H.Y., Elmali M., Karagöz A. (2015). Detection of Coxiella burnetii in cows’, goats’, and ewes’ bulk milk samples using polymerase chain reaction (PCR). Mljekarstvo.

[18] Jodelko A., Szymanska-Czerwinska M., Rola J.G., Niemczuk K. (2021). Molecular detection of Coxiella burnetii in small ruminants and genotyping of specimens collected from goats in Poland. BMC Vet. Res..

[19] Basanisi M.G., La Bella G., Nobili G., Raele D.A., Cafiero M.A., Coppola R., Damato A.M., Fraccalvieri R., Sottili R., La Salandra G. (2022). Detection of Coxiella burnetii DNA in sheep and goat milk and dairy products by droplet digital PCR in south Italy. Int. J. Food Microbiol..

[20] Simeonov K.B., Genova-Kalou P.D. (2023). Coxiella burnetii occurrence in dairy herds in Gabrovo Region, Bulgaria, evaluated by serological and molecular analyses of bulk-tank milk samples. Vet. Arhiv..

[21] Pires H., Santos-Silva S., Cruz A.V.S., Cardoso L., Lopes A.P., Pereira M.A., Nóbrega C., Mega A.C., Santos C., Cruz R. (2024). Molecular evidence of sporadic *Coxiella burnetii* excretion in sheep milk, central Portugal. Vet. Res. Comm..

[22] Hadjigeorgiou I., Vallerand F., Tsimpoukas K., Zervas G. (2002). The socio-economics of sheep and goat farming in Greece and the implications for future rural development. Opt. Méditerran. B Etud. Recherch..

[23] Pulina G., Milan M.J., Lavin M.P., Theodoridis A., Morin E., Capote J., Thomas D.L., Francesconi A.H.D., Caja G. (2018). Current production trends, farm structures, and economics of the dairy sheep and goat sector. J. Dairy Sci..

[24] Hellenic Statistical Authority (2022). Farm Structure Surveys. https://www.statistics.gr.

[25] Hellenic Agricultural Organization ‘Demeter’ (2024). Delivered Quantities and Number of Producers of Sheep/Goat Milk for the Year 2023. https://www.elgo.gr/images/ELOGAK_files/Statistics/ELGO_STATS/1.ΕΛΓO_STATS_ΠAΡAΔ_ΠOΣ__ΠAΡAΓ_ΠΡOΒΕΙOΥ_ΓΙΔΙΝO_AΝA_ΝOΜO__ΜHΝA_2023.pdf.

[26] Ministry of Agriculture, Hellenic Republic (1994). Recognition of protected designation of origin of cheese ‘Feta’. Ministerial decree 313025/11.01.1994. Governm. Gaz. Hell. Repub. B.

[27] Anon (2024). Increase in Feta Exports. https://www.enikonomia.gr/economy/afxisi-stis-exagoges-fetas-ti-deichnou/574665/.

[28] Ministry of Agricultural Development and Food (2019). Specifications for Arseniko Naxou Cheese. https://www.minagric.gr/images/stories/docs/agrotis/POP-PGE/prodiagrafes_arsenikou_naxou.pdf.

[29] Maltezou H.C., Constantopoulou I., Kallergi C., Vlahou V., Georgakopoulos D., Kafetzis D.A., Raoult D. (2004). Q fever in children in Greece. Am. J. Trop. Med. Hyg..

[30] European Food Safety Authority and European Centre for Disease Prevention and Control (2021). The European Union One Health 2019 zoonoses report. EFSA J..

[31] Lianou D.T. (2023). Mapping the Small Ruminant Industry in Greece: Health Management and Diseases of Animals, Preventive Veterinary Medicine and Therapeutics, Reproductive Performance, Production Outcomes, Veterinary Public Health, Socio-demographic Characteristics of the Farmers. Ph.D. Thesis.

[32] Satter S. (2024). Structured Questionnaire: Definition, Types + Pros & Cons. https://www.questionpro.com/blog/structured-questionnaire/.

[33] European Food Safety Authority (2014). Scientific opinion on the welfare risks related to the farming of sheep for wool, meat and milk production. EFSA J..

[34] Valiakos G., Giannakopoulos A., Spanos S.A., Korbou F., Chatzopoulos D.C., Mavrogianni V.S., Spyrou V., Fthenakis G.C., Billinis C. (2017). Use of geographical information system and ecological niche model to analyse potential exposure of small ruminants to *Coxiella burnetii* infection in Central Greece. Small Rumin. Res..

[35] Rodolakis A., Berri M., Hechard C., Caudron C., Souriau A., Bodier C., Blanchard B., Camuset P., Devillechaise P., Natorp J. (2007). Comparison of *Coxiella burnetii* shedding in milk of dairy bovine, caprine, and ovine herds. J. Dairy Sci..

[36] Bauer B., Prüfer L., Walter M., Ganter I., Frangoulidis D., Runge M., Ganter M. (2020). Comparison of *C. burnetii* excretion between sheep and goats naturally infected with one cattle-associated genotype. Pathogens.

[37] Sargison N. (2008). Sheep Flock Health A Planned Approach.

[38] Linklater K.A., Martin W.B., Aitken I.D. (2000). Other infectious causes of abortion. Diseases of Sheep.

[39] National Animal Disease Information Service (2022). Q Fever in Sheep. https://www.nadis.org.uk/disease-a-z/sheep/q-fever-in-sheep/.

[40] Plummer P.J., Winter A.L. (2022). Coxiellosis in animals. MSD Manual Veterinary Manual.

[41] World Organisation for Animal Health (2023). Q Fever. https://www.woah.org/en/disease/q-fever/#:~:text=is%20Q%20fever%3F-,Q%20fever%20is%20a%20widespread%20disease%20caused%20by%20the%20bacteria,animals%20that%20can%20infect%20humans.

[42] Nusinovici S., Madouasse A., Hoch T., Guatteo R., Beaudeau F. (2015). Evaluation of two PCR tests for *Coxiella burnetii* detection in dairy cattle farms using latent class analysis. PLoS ONE.

[43] Warrier I., Hicks L.D., Battisti J.M., Raghavan R., Minnick M.F. (2014). Identification of novel small RNAs and characterization of the 6S RNA of *Coxiella burnetii*. PLoS ONE.

[44] Ledda S., Santucciu C., Chisu V., Masala G. (2020). Validation of a novel commercial ELISA test for the detection of antibodies against *Coxiella burnetii*. Pathogens.

[45] Frangoulidis D., Meyer H., Kahlhofer C., Splettstoesser W.D. (2012). ‘Real-time’ PCR based detection of *Coxiella burnetii* using conventional techniques. FEMS Immunol. Med. Microbiol..

[46] Bustin S.A., Benes V., Garson J.A., Hellemans J., Huggett J., Kubista M., Mueller R., Nolan T., Pfaffl M.W., Shipley G.L. (2009). The MIQE Guidelines: Minimum information for publication of quantitative real-time PCR experiments. Clin. Chem..

[47] Panning M., Kilwinski J., Greiner-Fischer S., Peters M., Kramme S., Frangoulidis D., Meyer H., Henning K., Drosten C. (2008). High throughput detection of *Coxiella burnetii* by real-time PCR with internal control system and automated DNA preparation. BMC Microbiol..

[48] Khademi P., Tukmechi A., Sgroi G., Ownagh A., Enferadi A., Khalili M., Mardani K. (2024). Molecular and genotyping techniques in diagnosis of *Coxiella burnetii*: An overview. Inf. Gen. Evol..

[49] Roest H.J., van Gelderen B., Dinkla A., Frangoulidis D., van Zijderveld F.G., Rebel J., van Keulen L. (2012). Q fever in pregnant goats: Pathogenesis and excretion of *Coxiella burnetii*. PLoS ONE.

[50] Plummer P.J., McClure J.T., Menzies P., Morley P.S., Van den Brom R., Van Metre D.C. (2018). Management of *Coxiella burnetii* infection in livestock populations and the associated zoonotic risk: A consensus statement. J. Vet. Intern. Med..

[51] Agerholm J.S. (2013). *Coxiella burnetii* associated reproductive disorders in domestic animals—A critical review. Acta Vet. Scand..

[52] Seo M.G., Ouh I.O., Lee S.H., Kwak D. (2016). Detection and genotyping of *Coxiella burnetii* in pigs, South Korea, 2014–2015. Emerg. Infect. Dis..

[53] Celina S.S., Cerný J. (2022). *Coxiella burnetii* in ticks, livestock, pets and wildlife: A mini-review. Fr. Vet. Sci..

[54] Ferrara G., Pagnini U., Improda E., Iovane G., Montagnaro S. (2024). Pigs in southern Italy are exposed to three ruminant pathogens: An analysis of seroprevalence and risk factors analysis study. BMC Vet. Res..

[55] Apanaskevich D.A., Horak I.G., Mulumba-Mfumu L.K. (2013). A new species of *Rhipicephalus* (*Acari*: *Ixodidae*), a parasite of Red River hogs and domestic pigs in the Democratic Republic of Congo. J. Med. Entomol..

[56] Zhang Y.K., Zhang X.Y., Liu J.Z. (2019). Ticks (Acari: Ixodoidea) in China: Geographical distribution, host diversity, and specificity. Arch. Insect Biochem. Physiol..

[57] Melis S., Batisti Biffignandi G., Olivieri E., Galon C., Vicari N., Prati P., Moutailler S., Sassera D., Castelli M. (2024). High-throughput screening of pathogens in *Ixodes ricinus* removed from hosts in Lombardy, northern Italy. Ticks Tick Borne Dis..

[58] Emery M.P., Ostlund E.N., Schmitt B.J. (2012). Comparison of Q fever serology methods in cattle, goats, and sheep. J. Vet. Diagn..

[59] Karagul M.S., Malal M.E., Akar K. (2019). Seroprevalence of Q fever in sheep and goats from the Marmara region, Turkey. J. Vet. Res..

[60] De la Rua-Domenech R. (2006). Human *Mycobacterium bovis* infection in the United Kingdom: Incidence, risks, control measures and review of the zoonotic aspects of bovine tuberculosis. Tuberculosis.

[61] Langer A.J., Ayers T., Grass J., Lynch M., Angulo F.J., Mahonet B.E. (2012). Nonpasteurized dairy products, disease outbreaks, and state laws-United States, 1993–2006. Emerg. Inf. Dis..

[62] Wittwer M., Hammer P., Runge M., Valentin-Weigand P., Neubauer H., Henning K., Mertens-Scholz K. (2022). Inactivation kinetics of *Coxiella burnetii* during high-temperature short-time pasteurization of milk. Front. Microbiol..

[63] Enright J.B., Sadler W.W., Thomas R.C. (1957). Pasteurization of milk containing the organism of Q fever. Am. J. Pub. Health.

[64] Cutler S., Smithers G.W. (2023). Natural resistance of *Coxiella*. Encyclopedia of Food Safety.

[65] Cerf O., Condron R. (2006). *Coxiella burnetii* and milk pasteurization: An early application of the precautionary principle?. Epidemiol. Infect..

