# Supplementary material for: Detection of Coxiella burnetii in Bulk Tank Milk of Dairy Small Ruminant Farms in Greece
Source: Foods. 2025 Jan 31;14(3):460. doi: 10.3390/foods14030460 (PMC11817811; doi:10.3390/foods14030460)
Supplement: Supplementary file 1 [file foods-14-00460-s001.zip › foods-3379600-supplementary.pdf]

Detection of *Coxiella burnetii* in the bulk-tank milk of dairy small ruminant farms in Greece

D.T. Lianou, T. Giannoulis, C.K. Michael, N.G.C. Vasileiou, E. Petinaki, A.I. Katsafadou, A.P. Politis, D.A. Gougoulis, V.G. Papatsiros, E. Papadopoulos, E.I. Katsarou, V.S. Mavrogianni*, D.C. Chatzopoulos, G.C. Fthenakis

Table S1. Prevalence of detection of genetic material of *Coxiella burnetii* in the bulk milk tank of sheep and goat farms in accordance with published studies from countries around the world.

Country	Prevalence (no. of farms in the study)	Publication describing the study
		Sheep farms
Bulgaria	7.7% (*n* = 39)	Simeonov, K.B.; Genova-Kalou, P.D. *Coxiella burnetii* occurrence in dairy herds in Gabrovo Region, Bulgaria, evaluated by serological and molecular analyses of bulk-tank milk samples. *Veterinarski Arhiv* **2023**, *93*, 495-502.
Iran	0.0% (*n* = 110)	Cited by<br>Van den Brom, R.; van Engelen, E.; Roest, H.I.; van der Hoek, W.; Vellema, P. *Coxiella burnetii* infections in sheep or goats: An opinionated review. *Vet. Microbiol.* **2015**, *181*, 119–129.
Iran	6.0% (*n* = 140)	Cited by<br>Van den Brom, R.; van Engelen, E.; Roest, H.I.; van der Hoek, W.; Vellema, P. *Coxiella burnetii* infections in sheep or goats: An opinionated review. *Vet. Microbiol.* **2015**, *181*, 119–129.
Italy	23.9% (*n* = 285)	Basanisi, M.G.; La Bella, G.; Nobili, G.; Raele, D.A.; Cafiero, M.A.; Coppola, R.; Damato, A.M.; Fraccalvieri, R.; Sottili, R.; La Salandra, G. Detection of *Coxiella burnetii* DNA in sheep and goat milk and dairy products by droplet digital PCR in south Italy. *Int. J. Food Microbiol.* **2022**, *366*, 109583.
Poland	22.2% (*n* = 9)	Jodelko, A.; Szymanska-Czerwinska, M.; Rola, J.G.; Niemczuk, K. Molecular detection of *Coxiella burnetii* in small ruminants and genotyping of specimens collected from goats in Poland. *BMC Vet. Res.* **2021**, *17*, 341.
Portugal	1.3% (*n* = 78)	Pires, H.; Santos-Silva, S.; Cruz, A.V.S.; Cardoso, L.; Lopes, A.P.; Pereira, M.A.; Nóbrega, C.; Mega, A.C.; Santos, C.; Cruz, R.; Esteves, F.; Vala, H.; Matos, A.C.; Barradas, P.F.; Coelho, A.C.; Mesquita, J.R. Molecular evidence of sporadic *Coxiella burnetii* excretion in sheep milk, central Portugal. *Vet. Res. Comm.* **2024**, *48*, 2713-2719.
Spain	22.0% (*n* = 154)	García-Pérez, A.L.; Astobiza, I.; Barandika, J.F.; Atxaerandio, R.; Hurtado, A.; Juste, R.A. Investigation of *Coxiella burnetii* occurrence in dairy sheep flocks by bulk-tank milk analysis and antibody level determination. *J. Dairy Sci.* **2009**, *92*, 1581-1584.

Switzerland	0.0% (*n* = 81)	Fretz, R.; Schaeren, W.; Tanner, M.; Baumgartner, A. Screening of various foodstuffs for occurrence of Coxiella burnetii in Switzerland. *Int. J. Food Microbiol.* **2007**, *116*, 414-418.
Turkey	16.7% (*n* = 6)	Can, H.Y.; Elmali, M.; Karagöz, A. Detection of *Coxiella burnetii* in cows', goats', and ewes' bulk milk samples using polymerase chain reaction (PCR). *Mljekarstvo* **2015**, *65*, 26-31.
	Goat farms	
Bulgaria	0.0% (*n* = 9)	Simeonov, K.B.; Genova-Kalou, P.D. *Coxiella burnetii* occurrence in dairy herds in Gabrovo Region, Bulgaria, evaluated by serological and molecular analyses of bulk-tank milk samples. *Veterinarski Arhiv* **2023**, *93*, 495-502.
Iran	2.0% (*n* = 56)	Cited by Van den Brom, R.; van Engelen, E.; Roest, H.I.; van der Hoek, W.; Vellema, P. *Coxiella burnetii* infections in sheep or goats: An opinionated review. *Vet. Microbiol.* **2015**, *181*, 119–129.
Iran	5.0% (*n* = 110)	Cited by Van den Brom, R.; van Engelen, E.; Roest, H.I.; van der Hoek, W.; Vellema, P. *Coxiella burnetii* infections in sheep or goats: An opinionated review. *Vet. Microbiol.* **2015**, *181*, 119–129.
Poland	51.2% (*n* = 43)	Jodelko, A.; Szymanska-Czerwinska, M.; Rola, J.G.; Niemczuk, K. Molecular detection of *Coxiella burnetii* in small ruminants and genotyping of specimens collected from goats in Poland. *BMC Vet. Res.* **2021**, *17*, 341.
Switzerland	0.0% (*n* = 39)	Fretz, R.; Schaeren, W.; Tanner, M.; Baumgartner, A. Screening of various foodstuffs for occurrence of Coxiella burnetii in Switzerland. *Int. J. Food Microbiol.* **2007**, *116*, 414-418.
The Netherlands	33.0% (*n* = 292)	Van den Brom, R.; van Engelen, E.; Luttikholt, S.; Moll, L.; van Maanen, K.; Vellema, P. *Coxiella burnetii* in bulk tank milk samples from dairy goat and dairy sheep farms in The Netherlands in 2008. *Vet. Rec.* **2012**, *170*, 310.
Turkey	25.0% (*n* = 4)	Can, H.Y.; Elmali, M.; Karagöz, A. Detection of *Coxiella burnetii* in cows', goats', and ewes' bulk milk samples using polymerase chain reaction (PCR). *Mljekarstvo* **2015**, *65*, 26-31.

Table S2. List of variables evaluated for potential association with the detection of genetic material of *Coxiella burnetii* in the bulk-tank milk of 325 sheep flocks and 119 goat herds in Greece.

Variables related to management system applied in farms
Management system applied in farm (EFSA classification: shepherding / intensive / semi-intensive / semi-extensive / extensive / very extensive / mixed)
Variables related to infrastructure in farms
Availability of milking parlour (yes / no)
Availability of a main building for animals (yes / no)
Availability of accessory building(s) for animals (yes / no)
Availability of a dedicated building for lambs / kids (yes / no)
Availability of a dedicated lambing / kidding area (yes / no)
Variables related to animals in farms
No. of ewes / does on farms (no.)
Breed of animals (description)
Presence of cattle in the farm (yes / no)
Presence of dogs in the farm (yes / no)
Presence of cats in the farm (yes / no)
Presence of pigs in the farm (yes / no)
Presence of equines in the farm (yes / no)
Variables related to production characteristicis in farms
Month of the start of the lambing / kidding season (description)
Total milk quantity obtained per ewe / doe during the preceding milking period (litres)
Average number of lambs / kids born per ewe / doe during the preceding lambing / kidding season (no.)
Variables related to health management in farms
Collaboration with a veterinarian (yes / no)
Total visits made annually by veterinarians to the farm during the preceding season (no.)
Use of laboratory diagnostic examinations in samples of milk (yes / no)
Maintenance of quarantine period for new animals into the farm (yes / no)
Beginning of the mating period for female animals on the farm (month)
Application of reproductive management (yes / no)
Age of newborn removal from their dams (days)
Average age of culling female animals (years)
Daily number of milking sessions (no.)
Use of teat disinfection after milking (yes / no)
Administration of 'dry-ewe' treatment at the end of the lactation period (yes / no)
Duration of the dry-period (months)
Administration of oxytetracycline to the pregnant animals (yes / no)
Administration of selenium to pregnant animals (yes / no)
Common grazing of sheep / goats with wildlife ruminants (yes / no)
Seasonal transfer of animals to other site (yes / no)
Annual frequency of systemic disinfections in the farm (no. of occasions)
Provision of hay as fodder to animals (yes / no)
Quantity of hay provided per animal daily (kg)

Provision of straw to animals (yes / no)
Provision of silage to adult animals (yes / no)
Provision of finished feed (concentrate) to animals (yes / no)
Provision of finished feed (concentrate) to animals throughout the year (yes / no)
Variables related to human resources in farms
Length of previous animal farming experience (years)
Highest general education level achieved (primary / secondary / tertiary)
Farmer by profession (yes / no)
Daily period of presence at the farm (hours)
Family tradition in farming (yes / no)
Presence of working staff at the farm (yes / no)

Table S3. Details of multivariable models employed for the evaluation of potential associations with the detection of genetic material of *Coxiella burnetii* in the bulk-tank milk of 325 sheep flocks and 119 goat herds in Greece.

Outcome	Variables		
	**assessed in univariable analyses (*n*)**	**offered to the multi-variable models (*n*)**	**required in the final models**
Detection of genetic material of *C. burnetii* in the bulk-tank milk from sheep farms	47	7	(a) Management system applied in farm, (b) Availability of a main building for animals, (c) Availability of accessory building(s) for animals, (d) Seasonal transfer of animals to other site, (e) Provision of silage to adult animals
Detection of genetic material of *C. burnetii* in the bulk-tank milk from goat farms	47	10	(a) Management system applied in farm, (b) Availability of a dedicated kidding area, (c) No. of does on farms, (d) Presence of equines in the farm, (e) Total visits made annually by veterinarians to the farm
Detection of genetic material of *C. burnetii* in the bulk-tank milk from sheep farms under intensive or semi-intensive management	46	6	(a) Availability of accessory building(s) for animals, (b) Availability of a dedicated building for lambs (c) Average age of culling female animals, (d) Seasonal transfer of animals to other site, (e) Provision of silage to adult animals
Detection of genetic material of *C. burnetii* in the bulk-tank milk from goat farms under intensive or semi-intensive management	46	11	(a) Presence of pigs in the farm, (b) Presence of equines in the farm

Table S4. Results of univariable analysis for the detection of genetic material of *Coxiella burnetii* in the bulk-tank milk of 325 sheep flocks in Greece.

Variables	r_{sp}	*p* value
Management system applied in farm	0.110	0.048
Availability of milking parlour	0.043	0.44
Availability of a main building for animals	–0.104	0.06
Availability of accessory building(s) for animals	–0.087	0.12
Availability of a dedicated building for lambs	0.055	0.32
Availability of a dedicated lambing area	0.008	0.89
No. of ewes on farms	–0.010	0.86
Breed of animals	0.006	0.92
Presence of cattle in the farm	–0.057	0.31
Presence of dogs in the farm	0.051	0.36
Presence of cats in the farm	–0.009	0.87
Presence of pigs in the farm	0.060	0.28
Presence of equines in the farm	0.070	0.21
Month of the start of the lambing season	0.096	0.08
Total milk quantity obtained per ewe during the preceding milking period	0.039	0.48
Average number of lambs born per ewe during the preceding lambing season	–0.012	0.83
Collaboration with a veterinarian	0.009	0.87
Total visits made annually by veterinarians to the farm during the preceding season	–0.048	0.38
Use of laboratory diagnostic examinations in samples of milk	0.003	0.96
Maintenance of quarantine period for new animals into the farm	0.006	0.91
Beginning of the mating period for female animals on the farm	0.044	0.43
Application of reproductive management	0.001	0.98
Age of newborn removal from their dams	0.030	0.59
Average age of culling female animals	0.112	0.044
Daily number of milking sessions	0.017	0.76
Use of teat disinfection after milking	0.029	0.61
Administration of 'dry-ewe' treatment at the end of the lactation period	0.024	0.67
Duration of the dry-period	0.002	0.97
Administration of oxytetracycline to the pregnant animals	0.026	0.65
Administration of selenium to pregnant animals	0.013	0.82
Common grazing of sheep with wildlife ruminants	0.011	0.84
Seasonal transfer of animals to other site	0.086	0.12
Annual frequency of systemic disinfections in the farm	0.024	0.67
Provision of hay as fodder to animals	0.009	0.87
Quantity of hay provided per animal daily	–0.046	0.40
Provision of straw to animals	–0.007	0.90
Provision of silage to adult animals	–0.090	0.11
Provision of finished feed to animals	0.019	0.74
Provision of finished feed (concentrate) to animals throughout the year	–0.003	0.96
Length of previous animal farming experience	–0.024	0.67

Highest general education level achieved	–0.054	0.33
Farmer by profession	–0.005	0.92
Daily period of presence at the farm	0.032	0.56
Family tradition in farming	–0.047	0.40
Presence of working staff at the farm	0.023	0.68

Table S5. Results of univariable analysis for the detection of genetic material of *Coxiella burnetii* in the bulk-tank milk of 119 goat herds in Greece.

Variables	r_{sp}	*p* value
Management system applied in farm	–0.254	0.005
Availability of milking parlour	–0.025	0.78
Availability of a main building for animals	0.030	0.75
Availability of accessory building(s) for animals	–0.064	0.49
Availability of a dedicated building for kids	0.057	0.54
Availability of a dedicated kidding area	–0.148	0.11
No. of does on farms	–0.145	0.12
Breed of animals	0.139	0.13
Presence of cattle in the farm	0.028	0.76
Presence of dogs in the farm	0.043	0.64
Presence of cats in the farm	0.079	0.39
Presence of pigs in the farm	0.154	0.09
Presence of equines in the farm	–0.119	0.19
Month of the start of the kidding season	–0.045	0.63
Total milk quantity obtained per doe during the preceding milking period	0.017	0.86
Average number of kids born per doe during the preceding kidding season	0.091	0.33
Collaboration with a veterinarian	–0.010	0.91
Total visits made annually by veterinarians to the farm during the preceding season	–0.209	0.022
Use of laboratory diagnostic examinations in samples of milk	–0.119	0.19
Maintenance of quarantine period for new animals into the farm	0.071	0.44
Beginning of the mating period for female animals on the farm	0.058	0.53
Application of reproductive management	–0.001	0.99
Age of newborn removal from their dams	–0.125	0.18
Average age of culling female animals	–0.118	0.21
Daily number of milking sessions	0.108	0.24
Use of teat disinfection after milking	–0.077	0.40
Administration of 'dry-ewe' treatment at the end of the lactation period	–0.088	0.34
Duration of the dry-period	–0.063	0.50
Administration of oxytetracycline to the pregnant animals	–0.079	0.39
Administration of selenium to pregnant animals	0.012	0.90
Common grazing of goats with wildlife ruminants	0.059	0.52
Seasonal transfer of animals to other site	0.053	0.57
Annual frequency of systemic disinfections in the farm	0.042	0.65
Provision of hay as fodder to animals	0.037	0.69
Quantity of hay provided per animal daily	–0.070	0.45
Provision of straw to animals	–0.054	0.56
Provision of silage to adult animals	–0.097	0.29
Provision of finished feed to animals	–0.208	0.023
Provision of finished feed (concentrate) to animals throughout the year	–0.041	0.66
Length of previous animal farming experience	0.058	0.53

Highest general education level achieved	–0.116	0.21
Farmer by profession	–0.035	0.71
Daily period of presence at the farm	–0.051	0.58
Family tradition in farming	0.091	0.33
Presence of working staff at the farm	0.024	0.79

Table S6. Results of univariable analysis for the detection of genetic material of *Coxiella burnetii* in the bulk-tank milk of farms under intensive or semi-intensive management among 325 sheep flocks in Greece.

Variables	r_{sp}	*p* value
Availability of milking parlour	0.059	0.43
Availability of a main building for animals	0.016	0.83
Availability of accessory building(s) for animals	0.112	0.13
Availability of a dedicated building for lambs	–0.134	0.07
Availability of a dedicated lambing area	0.019	0.80
No. of ewes on farms	–0.046	0.54
Breed of animals	0.005	0.95
Presence of cattle in the farm	–0.081	0.28
Presence of dogs in the farm	0.049	0.51
Presence of cats in the farm	0.003	0.97
Presence of pigs in the farm	–0.081	0.28
Presence of equines in the farm	0.081	0.27
Month of the start of the lambing season	0.122	0.10
Total milk quantity obtained per ewe during the preceding milking period	0.039	0.60
Average number of lambs born per ewe during the preceding lambing season	0.004	0.96
Collaboration with a veterinarian	0.068	0.36
Total visits made annually by veterinarians to the farm during the preceding season	–0.062	0.40
Use of laboratory diagnostic examinations in samples of milk	0.003	0.97
Maintenance of quarantine period for new animals into the farm	0.018	0.81
Beginning of the mating period for female animals on the farm	–0.043	0.56
Application of reproductive management	–0.002	0.97
Age of newborn removal from their dams	0.001	0.99
Average age of culling female animals	0.117	0.12
Daily number of milking sessions	0.014	0.85
Use of teat disinfection after milking	0.020	0.79
Administration of 'dry-ewe' treatment at the end of the lactation period	–0.057	0.44
Duration of the dry-period	0.031	0.68
Administration of oxytetracycline to the pregnant animals	0.044	0.55
Administration of selenium to pregnant animals	0.010	0.90
Common grazing of sheep with wildlife ruminants	0.029	0.70
Seasonal transfer of animals to other site	0.123	0.10
Annual frequency of systemic disinfections in the farm	0.028	0.70
Provision of hay as fodder to animals	0.016	0.83
Quantity of hay provided per animal daily	–0.078	0.29
Provision of straw to animals	–0.004	0.96
Provision of silage to adult animals	–0.130	0.08
Provision of finished feed to animals	0.016	0.83
Provision of finished feed (concentrate) to animals throughout the year	–0.043	0.56
Length of previous animal farming experience	0.020	0.79
Highest general education level achieved	–0.087	0.24

Farmer by profession	–0.015	0.84
Daily period of presence at the farm	0.089	0.23
Family tradition in farming	–0.039	0.60
Presence of working staff at the farm	0.002	0.98

Table S7. Results of univariable analysis for the detection of genetic material of *Coxiella burnetii* in the bulk-tank milk of farms under intensive or semi-intensive management among 119 goat herds in Greece.

Variables	r_{sp}	*p* value
Availability of milking parlour	–0.181	0.28
Availability of a main building for animals	n/r	n/r
Availability of accessory building(s) for animals	0.010	0.95
Availability of a dedicated building for kids	0.249	0.13
Availability of a dedicated kidding area	–0.276	0.09
No. of does on farms	–0.266	0.11
Breed of animals	0.171	0.30
Presence of cattle in the farm	–0.034	0.84
Presence of dogs in the farm	0.064	0.70
Presence of cats in the farm	0.185	0.27
Presence of pigs in the farm	0.258	0.12
Presence of equines in the farm	–0.233	0.16
Month of the start of the kidding season	0.022	0.90
Total milk quantity obtained per doe during the preceding milking period	–0.085	0.61
Average number of kids born per doe during the preceding kidding season	0.114	0.50
Collaboration with a veterinarian	0.092	0.58
Total visits made annually by veterinarians to the farm during the preceding season	–0.315	0.05
Use of laboratory diagnostic examinations in samples of milk	–0.233	0.16
Maintenance of quarantine period for new animals into the farm	0.194	0.24
Beginning of the mating period for female animals on the farm	0.133	0.43
Application of reproductive management	–0.034	0.84
Age of newborn removal from their dams	–0.090	0.59
Average age of culling female animals	–0.090	0.59
Daily number of milking sessions	0.079	0.64
Use of teat disinfection after milking	–0.185	0.27
Administration of ‘dry-ewe’ treatment at the end of the lactation period	–0.233	0.16
Duration of the dry-period	0.130	0.44
Administration of oxytetracycline to the pregnant animals	–0.057	0.73
Administration of selenium to pregnant animals	0.048	0.78
Common grazing of goats with wildlife ruminants	–0.114	0.50
Seasonal transfer of animals to other site	0.045	0.79
Annual frequency of systemic disinfections in the farm	–0.151	0.37
Provision of hay as fodder to animals	n/r	n/r
Quantity of hay provided per animal daily	–0.231	0.16
Provision of straw to animals	–0.217	0.19
Provision of silage to adult animals	–0.281	0.09
Provision of finished feed to animals	n/r	n/r
Provision of finished feed (concentrate) to animals throughout the year	–0.121	0.47
Length of previous animal farming experience	0.120	0.47
Highest general education level achieved	–0.152	0.36

Farmer by profession	–0.079	0.64
Daily period of presence at the farm	–0.037	0.83
Family tradition in farming	0.185	0.27
Presence of working staff at the farm	–0.037	0.83

Table S8. Production-related outcomes [1] in farms where *Coxiella burnetii* was or was not detected in the bulk-tank milk among 325 sheep flocks and 119 goat herds in Greece.

Production-related outcomes	Farms where genetic material of *C. burnetii* was not detected in the bulk-tank milk	Farms where genetic material of *C. burnetii* was detected in the bulk-tank milk	*p* value
Sheep farms			
Total milk quantity obtained per ewe	198 (125) L	200 (59) L	0.48
Average number of lambs born per ewe	1.3 (0.2)	1.3 (0.3)	0.83
Somatic cell counts in bulk-tank milk	0.498×10^6 (0.462×10^6) cells mL^{-1}	0.636×10^6 (0.401×10^6) cells mL^{-1}	0.83
Fat content in bulk-tank milk	6.2% (1.0%)	6.2% (0.7%)	0.94
Protein content in bulk-tank milk	4.4% (0.3%)	4.4% (0.4%)	0.83
Goat farms			
Total milk quantity obtained per ewe	160 (136) L	155 (70) L	0.86
Average number of lambs born per ewe	1.3 (0.2)	1.3 (0.1)	0.33
Somatic cell counts in bulk-tank milk	0.899×10^6 (0.650×10^6) cells mL^{-1}	0.755×10^6 (0.516×10^6) cells mL^{-1}	0.45
Fat content in bulk-tank milk	4.5% (1.6%)	5.1% (0.9%)	0.49
Protein content in bulk-tank milk	3.1% (0.4%)	3.1% (0.5%)	0.55

[1] median (interquartile range).

Table S9. Health-related outcomes in farms where *Coxiella burnetii* was or was not detected in the bulk-tank milk among 325 sheep flocks and 119 goat herds in Greece.

Health-related outcomes	Farms where genetic material of *C. burnetii* was not detected in the bulk-tank milk	Farms where genetic material of *C. burnetii* was detected in the bulk-tank milk	*p* value
Sheep farms			
Annual incidence of abortion cases	0.0% (2.8%)	0.0% (1.7%)	0.76
Proportion of farmers who declared abortion as significant health problem among replacement animals	3.8% (2.2%-6.5%)	0.0% (0.0%-29.9%)	0.55
Proportion of farmers who declared abortion as significant health problem among adult animals	5.1% (3.1%-8.1%)	11.1% (2.0%-43.5%)	0.42
Goat farms			
Annual incidence of abortion cases	0.0% (3.6%)	1.0% (4.7%)	0.93
Proportion of farmers who declared abortion as significant health problem among replacement animals	6.2% (3.0%-12.2%)	0.0% (0.0%-32.4%)	0.47
Proportion of farmers who declared abortion as significant health problem among adult animals	3.5% (1.4%-8.8%	0.0% (0.0%-32.4%)	0.59

[1] median (interquartile range) or proportion (95% confidence interval), as appropriate.
